# Diagnosis of Digital Ischemia During the COVID-19 Pandemic: A Study From a Developing Country

**DOI:** 10.7759/cureus.82459

**Published:** 2025-04-17

**Authors:** Bidhan Neupane, Md Nazrul Islam, Abul Khair Ahmedullah, Haner Direskeneli, Md Saif Ullah Khan

**Affiliations:** 1 Department of Medicine, Nepal Medical College, Kathmandu, NPL; 2 Department of Rheumatology, Bangabandhu Sheikh Mujib Medical University, Dhaka, BGD; 3 Department of Internal Medicine, Marmara University School of Medicine, Istanbul, TUR; 4 Department of Vascular Surgery, Bangabandhu Sheikh Mujib Medical University, Dhaka, BGD

**Keywords:** covid-19, diagnosis, digital ischemia, vasculitis, vasculopathy

## Abstract

Objective: Digital ischemia (DI) is an uncommon condition. Information on the etiology of DI is limited. This study aimed to determine the diagnosis of DI in a tertiary care center in Bangladesh.

Methods: This cross-sectional study was conducted in the rheumatology department of Bangabandhu Sheikh Mujib Medical University (BSMMU), Dhaka, Bangladesh, from September 1, 2020, to August 31, 2021. A total of 25 consecutive patients with DI were enrolled. Each patient was assessed following the classification/diagnostic criteria for vasculitis and vasculopathy. The 2012 International Chapel Hill Consensus Conference on the Nomenclature of Vasculitides was used for case definition. The study subjects were divided into vasculitis and vasculopathy groups. Fisher’s exact and Student’s t-tests were used to compare the groups.

Results: The mean age was 35.88 years with a female predominance (72%). Among 25 patients, 15 (60%) were in the vasculitis group and 10 (40%) in the vasculopathy group. Takayasu arteritis was found in one subject (4%), and another 14 had vasculitis associated with connective tissue diseases: 11 (44%) lupus vasculitis, two (8%) rheumatoid vasculitis, and one (4%) associated with dermatomyositis. In the vasculopathy group, five cases (20%) had systemic sclerosis (SSc), four (16%) had peripheral arterial disease (PAD), and one (4%) had primary antiphospholipid syndrome (APS). Fever (p=0.01), arthralgia/arthritis (p=0.01), and high CRP (p=0.04) were significantly associated with vasculitis.

Conclusion: In the hospital setting, connective tissue disease-associated vasculitis and SSc-related vasculopathy were the common causes of DI.

## Introduction

Digital ischemia (DI) is defined clinically as painful digit/s with signs of ischemia or necrosis. The estimated incidence is 2/100,000. It has various etiologies, of which many are poorly understood [1]. The most reported causes are cardiac or arterial embolism, local thrombosis, autoimmune connective tissue diseases (CTDs) such as systemic sclerosis (SSc), vasculitis, or trauma [2].

Among the rheumatic diseases as a cause of limb ischemia, the highest prevalence was observed in patients with SSc and mixed connective tissue disease (MCTD), which approached 90% or greater [3]. The prevalence of Raynaud’s phenomenon (RP) in primary Sjögren’s syndrome (pSS) and systemic lupus erythematosus (SLE) ranged between 13-66% and 10-45%, respectively [4,5]. Up to 17% of patients with rheumatoid arthritis (RA) suffer from this condition [3]. In antiphospholipid syndrome (APS), DI was reported in 3.3-7.5% of cases [6]. Also, 20% of patients with dermatomyositis/polymyositis (DM/PM) and about 50% with undifferentiated connective tissue disease (UCTD) showed this manifestation [7,5]. DI of the upper extremity due to vasculitis was 3.1% and included giant cell arteritis (GCA), Takayasu arteritis (TAK), cryoglobulinemia, necrotizing small vessel vasculitis, and unclassifiable vasculitis. Other studies showed a prevalence of 0-12% [2].

Atherosclerotic peripheral arterial disease (PAD) was seen in 4.3-29% of adults and mainly involved the lower limbs [8,9]. It is an emerging issue in low- and middle-income countries due to increasing traditional cardiovascular risk factors [10]. Digital gangrene was present in more than 70% of patients with thromboangiitis obliterans (TAO) [11], but different studies showed variable distribution ranging from 0% to 22% [2]. DI associated with cancer (DIAC) was 12-15% [12], whereas other studies found up to 8% [2]. The prevalence was 0-33% for hypothenar hammer syndrome (HHS), 0-9.3% for drugs, 2.8% for trauma, 2.5% for thoracic outlet syndrome, 2.2% each for thrombophilia and infectious disease, 1.5% for vasospasm, and 0.9% for peripheral embolism [2].

So far, there are few studies on the etiology of DI and its prevalence. To our knowledge, information on DI from the Asia-Pacific region is limited and unavailable. To date, no study has been published from Bangladesh. Therefore, this study aimed to determine the diagnosis of DI at a tertiary rheumatology center in Bangladesh.

## Materials and methods

This observational cross-sectional study was conducted in the department of rheumatology of Bangabandhu Sheikh Mujib Medical University (BSMMU), Dhaka, Bangladesh, from September 1, 2020, to August 31, 2021. Both male and female patients ≥18 years of age with DI (RP and/or digital ulcer/necrosis), giving consent to participate in the study, were included. Patients ≥18 years and not giving consent or below 18 years of age were kept in the exclusion criteria. A total of 25 consecutive patients were enrolled after having informed written consent.

RP was defined clinically as a well-demarcated color change of digit(s). Each patient was evaluated with a thorough history and clinical examination. For body mass index (BMI), Asia-Pacific classification was followed. Investigations like reverse transcription-polymerase chain reaction (RT-PCR) for COVID-19, complete blood count (CBC), erythrocyte sedimentation rate (ESR), C-reactive protein (CRP), serum creatinine, alanine transaminase (ALT), blood sugar (fasting and two hours after breakfast), fasting lipid profile, urine routine and microscopic examination (RME), chest X-ray posteroanterior (PA) view, 12 lead electrocardiogram (ECG), and duplex ultrasonography study of involved limb(s), were sent in every patient. Other tests like glycated hemoglobin (HbA1c), anti-centromere antibody (anti-centromere Ab), anti-topoisomerase I antibody (anti-Scl 70 Ab), nail-fold capillaroscopy, echocardiography, high-resolution computed tomography (HRCT) of chest, anti-ribonucleoprotein antibody (anti-RNP Ab), antinuclear antibody (ANA), anti-double-stranded DNA antibody (anti-ds DNA Ab), anti-Smith antibody (anti-Smith Ab), complement component 3 (C3), complement component 4 (C4), lupus anticoagulant, anti-cardiolipin antibody (anti-cardiolipin Ab), anti-beta-2 glycoprotein 1 antibody (anti-β2 GP1 Ab), anti-Ro/Sjögren’s-syndrome-related antigen A antibody (anti-Ro/SSA Ab), anti-La/Sjögren’s-syndrome-related antigen B antibody (anti-La/SSB Ab), lip biopsy, rheumatoid factor (RF), anti-cyclic citrullinated peptide antibody (ACPA), creatine kinase (CK), aldolase, aspartate transaminase (AST), lactate dehydrogenase (LDH), electromyography (EMG), muscle biopsy, perinuclear anti-neutrophil cytoplasmic antibody (p-ANCA), cytoplasmic anti-neutrophil cytoplasmic antibody (c-ANCA), angiography, hepatitis B surface antigen (HBsAg), anti-hepatitis C virus antibody (anti-HCV), and blood culture and sensitivity (C/S) were requested when indicated. All investigations were performed in BSMMU.

Data were collected and recorded in a semi-structured datasheet (Appendix A). A flowchart was made for each patient with all the related information about the onset and course of illness. For diagnosis, established classification/diagnostic criteria for vasculitis and vasculopathy were used. Study subjects were divided into vasculitis and vasculopathy groups. The case was defined using the 2012 International Chapel Hill Consensus Conference on the Nomenclature of Vasculitides. DM, RA, and SLE were described as CTDs. In cases of overlap, the etiology was derived from the disease activity status (e.g., in the case of SLE and RA overlap, diagnosis of lupus vasculitis was made based on active SLE). Bias was tried to overcome by following the above-mentioned process. Data were entered in IBM SPSS Statistics for Windows, Version 26 (Released 2020; IBM Corp., Armonk, New York, United States), and the level of significance was analyzed between two groups using Fisher’s exact test for gender, smoking, oral contraceptive pill (OCP) use, constitutional features, and co-morbidities. Student’s t-test was used for age, BMI, disease duration, hemoglobin, WBC count, platelet count, ESR, CRP, serum creatinine, ALT, blood sugar (fasting and two hours after breakfast), total cholesterol, HDL cholesterol, LDL cholesterol, and triglyceride. The confidence interval (CI) was set at 95%. P-value <0.05 was considered significant.

## Results

There were 18 women (72%) and seven men (28%) with a female-to-male ratio of 2.6:1. The mean age was 35.88 ± 11.73 years (range, 20-60 years). Among the occupations, 16 cases (64%) were homemakers, seven (28%) others, and two (8%) unemployed. A single subject (4%) was illiterate. Only male patients were smokers. Among six smokers, two cases with PAD were current smokers, and the other four (one SLE, one RA, one DM, and one PAD) were ex-smokers. None had a history of substance abuse. Six women (four SLE, one PAS, and one SSc) used OCPs. The mean BMI was 21.67 ± 4.22 kg/m^2^; five (20%) were underweight, 13 (52%) normal, and seven (28%) were overweight and obese together.

None of the study subjects had a family history of sudden death, an autoimmune disease in first-degree relatives, recent trauma, or a positive COVID-19 test. The clinical and laboratory characteristics of study subjects are shown in Tables 1-2, respectively.

**Table 1:** Clinical characteristics of study subjects (n=25) SD: Standard deviation; IQR: Interquartile range

Variable	Number (%)
Disease duration in months (mean ± SD), median (IQR)	24.72 ± 30.78, 12 (2.50-42.00)
Constitutional features	
Fever	8 (32)
Arthralgia/arthritis	16 (64)
Myalgia	4 (16)
Weight loss	13 (52)
Co-morbidities	
Hypertension	6 (24)
Diabetes (including prediabetes)	9 (36)
Dyslipidemia	22 (88)
Coronary artery disease	2 (8)

**Table 2:** Laboratory characteristics of study subjects (n=25) SD: Standard deviation; IQR: Interquartile range; WBC: White blood cell; ESR: Erythrocyte sedimentation rate; CRP: C-reactive protein; ALT: Alanine aminotransferase; FBS: Fasting blood sugar; BS: Blood sugar; ABF: After breakfast; HDL: High density lipoprotein; LDL: Low density lipoprotein

Variable	Mean ± SD, median (IQR)	Reference range
Hemoglobin (g/dL)	11.54 ± 1.84	12-14 (female)/14-16 (male)
WBC count (/cumm)	9834 ± 4749.65	4000-11000
Platelet count (/cumm)	332400 ± 150552.32	150000-450000
ESR (mm in 1^st^ hour)	42.52 ± 34.98, 33 (25.00-44.00)	10 (male)/20 (female)
CRP (mg/L)	27.39 ± 46.55, 8.2 (3.07-23.07)	<6
Serum creatinine (mg/dL)	0.74 ± 0.19	0.6-1.2
ALT (U/L)	33.60 ± 32.99, 23 (17.00-41.00)	10-40
FBS (mmol/L)	6.27 ± 4.48, 4.8 (4.35-6.25)	3.9-6.1
BS 2 hours ABF (mmol/L)	8.80 ± 6.18, 6.6 (5.75-10.10)	7.8-11
Total cholesterol (mg/dL)	168.48 ± 55.14	<200
HDL cholesterol (mg/dL)	36.84 ± 13.36	>40
LDL cholesterol (mg/dL)	90.36 ± 31.00	<130
Triglyceride (mg/dL)	206.24 ± 196.26, 164 (116.00-223.00)	<150

The diagnoses of DI are shown in Table 3. Among all the cases, 84% had rheumatic conditions. The most common etiology was CTD (56%).

**Table 3:** Diagnosis of digital ischemia (n=25) ^∆^ Nine classic SLE, one SLE/RA overlap, one SLE/TAK overlap ^+^ Fulfilled classification criteria ^ǂ^ Four cases of systemic sclerosis fulfilled the classification criteria, and one was labeled as early scleroderma on the basis of Raynaud’s phenomenon, abnormal nail-fold capillaries (active scleroderma pattern), and positive antinuclear antibody. SLE: systemic lupus erythematosus; RA: rheumatoid arthritis; TAK: Takayasu arteritis

Nomenclature	n (%)	Diagnosis
Vasculitis (n=15) (based on 2012 International Chapel Hill Consensus Conference)		
Large-vessel vasculitis	1 (4)	Takayasu arteritis^+^
Medium-vessel vasculitis	0 (0)	
Small-vessel vasculitis	0 (0)	
Variable vessel vasculitis	0 (0)	
Single-organ vasculitis	0 (0)	
Vasculitis associated with systemic disease (n=14)	11 (44)	Lupus vasculitis^∆+^
	2 (8)	Rheumatoid vasculitis^+^
	1 (4)	Dermatomyositis vasculitis^+^
Vasculitis associated with probable etiology	0 (0)	
Vasculopathy (n=10)		
Systemic sclerosis^ǂ^	5 (20)	
Peripheral arterial disease	4 (16)	
Primary antiphospholipid syndrome	1 (4)	

The clinicodemographic and laboratory characteristics of the vasculitis and vasculopathy groups are shown in Tables 4-5, respectively.

**Table 4:** Comparison of clinicodemographic characteristics of vasculitis and vasculopathy groups (n=25) SD: Standard deviation; OCP: Oral contraceptive pill; BMI: Body mass index ^s^ Student's t-test; ^f^ Fisher’s exact test; * Statistically significant

Variable	Vasculitis (n=15)	Vasculopathy (n=10)	P-value
Age in years (mean ± SD)	35.27 ± 11.74	36.80 ± 12.29	0.76^s^
Gender			0.60^f^
Female	11	7	
Male	4	3	
Smokers	3	3	0.46^f^
OCP users	4	2	0.56^f^
BMI (mean ± SD)	21.08 ± 3.43	22.54 ± 5.26	0.41^s^
Underweight	4	1	
Normal	7	6	
Obese and overweight	4	3	
Disease duration in months (mean ± SD)	28.20 ± 36.85	19.50 ± 19.05	0.45^s^
Constitutional features	15	6	0.02^f^*
Fever	8	0	0.01^f^*
Arthralgia or arthritis	13	3	0.01^f^*
Myalgia	3	1	0.47^f^
Weight loss	9	4	0.28^f^
Co-morbidities	14	8	0.35^f^
Hypertension	3	3	0.46^f^
Diabetes (including prediabetes)	4	5	0.22^f^
Dyslipidemia	14	8	0.35^f^
Coronary artery disease	1	1	0.65^f^

**Table 5:** Comparison of laboratory characteristics of vasculitis and vasculopathy groups (n=25) SD: Standard deviation; WBC: White blood cell; ESR: Erythrocyte sedimentation rate; CRP: C-reactive protein; ALT: Alanine aminotransferase; FBS: Fasting blood sugar; BS: Blood sugar; ABF: After breakfast; HDL: High density lipoprotein; LDL: Low density lipoprotein ^s^ Student's t-test; * Statistically significant

Variable (unit)	Vasculitis (n=15), mean ± SD	Vasculopathy (n=10), mean ± SD	P-value^s^
Hemoglobin (g/dL)	11.05 ± 1.76	12.27 ± 1.80	0.11
WBC count (/cumm)	9534.67 ± 5809.24	10283.00 ± 2699.75	0.71
Platelet count (/cumm)	300533 ± 108276	370200 ± 189036	0.25
ESR (mm in 1^st^ hour)	44.80 ± 36.52	39.10 ± 34.17	0.70
CRP (mg/L)	40.26 ± 36.52	8.09 ± 5.97	0.04*
Serum creatinine (mg/dL)	0.77 ± 0.22	0.71 ± 0.15	0.48
ALT (U/L)	38.20 ± 40.95	26.70 ± 14.34	0.41
FBS (mmol/L)	6.60 ± 5.73	5.47 ± 1.09	0.55
BS 2 hours ABF (mmol/L)	8.88 ± 7.48	8.27 ± 3.53	0.81
Total cholesterol (mg/dL)	173.27 ± 66.76	161.30 ± 32.85	0.61
HDL cholesterol (mg/dL)	36.93 ± 15.05	36.70 ± 11.11	0.97
LDL cholesterol (mg/dL)	89.33 ± 35.18	91.90 ± 25.17	0.84
Triglyceride (mg/dL)	236.40 ± 247.64	161.00 ± 59.40	0.36

In our study, four cases (16%) were PAD (three men and one woman). Two male patients were less than 40 years of age. Two subjects, one male and one female, had upper limb involvement. In the male subgroup, the risk factors of PAD were smoking, obesity, hypertension, diabetes, and dyslipidemia in three, one, one, two, and three cases, respectively. The female patient with PAD had obesity, hypertension, and dyslipidemia.

## Discussion

DI may progress to a critical stage. A careful consideration of various etiologies during assessment is imperative. Early and correct diagnosis of DI is often challenging for clinicians. Due to overlapping features of several diseases, using classification/diagnostic criteria may help in quick assessment in resource-constrained settings.

In our study, most subjects (56%) had CTD. Raimbeau et al. found CTD as the most common cause (19%) of DI, including SSc (3.1%). Other studies have shown CTD as an etiology of limb ischemia in up to 49% of cases. The prevalence of CTD, except SSc, in those studies varied from 0% to 28%. Among nine published articles on DI, seven had shown CTD as the most common etiology [2]. The high prevalence of CTD in the present study may be due to our rheumatology referral center status, and the wide variation may be due to enrollment criteria.

The mean age of subjects with DI was 35.88 years (range, 20-60 years), with a female predominance (72%). Also, all cases of CTD were between 20 and 60 years of age. These observations were similar to those of Gaubitz [13] and Spagnolo et al. [14]. They have mentioned that CTD was common among women between 15 and 60 years of age.

Compared to the general population, traditional risk factors for atherosclerosis are higher in patients with CTD [15]. In our study, the rate of hypertension (24%) and diabetes (including prediabetes) (36%) was high, but among the CTD cases, 8% had hypertension and 16% had diabetes (including 4% prediabetes). A similar finding for diabetes (12%) but not for hypertension (28%) was reported from China [16]. The cause was unknown for the low prevalence of hypertension in the present study. In this study, 88% of total cases and 52% of subjects with CTD had dyslipidemia. Avinash et al. showed dyslipidemia in 80% of patients with CTD (including 27% SSc cases), comparable to our result [17]. Early identification of those risk factors in CTD patients and subsequent steps may reduce the complications.

In our study, out of 15 cases of vasculitis, a single case was TAK, and the others were vasculitis associated with CTDs. Among them, 11 (44%) were lupus vasculitis (nine classic SLE, one SLE/RA overlap, one SLE/TAK overlap), two (8%) rheumatoid vasculitis, and one (4%) DM vasculitis. Vasculitis occurred in around half of the patients with SLE [18], and lupus vasculitis was “probably the most common cause of vasculitis-associated digital ischemia” [11].

Among the vasculopathies, SSc (20%) was the most common cause of DI. A similar observation was noted by Raimbeau et al. [2]. All had RP as a presenting feature, a similar finding to that reported by Walker et al. [19].

The rate of PAD (16%) in this study was within the range mentioned by Weinberg and Jaff [8]. Fifty percent of our PAD cases were under 40 years of age and had DI in the upper limbs with several traditional risk factors. Similar findings were shown by Upganlawar et al., who disclosed that such an occurrence is possible in the presence of conventional risk factors of atherosclerosis [20].

In the present study, fever, arthralgia/arthritis, and high CRP favored vasculitis. CRP, one of the markers of inflammation, is used to rule out vasculitis [21] and is markedly elevated with active disease [22]. Constitutional symptoms and acute phase response may suggest vasculitis [11]. During a case evaluation, the presence of these markers may guide the diagnosis of vasculitis.

In this study, none had occupations requiring vibrating tools, though Cordeiro and Andrade mentioned such tools as one of the risk factors for DI [23]. We observed no correlation between education level and DI, similar to a study from Greece [24].

Strength of the study

This study to determine the diagnosis of DI using established classification/diagnostic criteria is the first in Bangladesh. Our study can help to develop a proposal for early diagnosis and initiation of treatment in resource-constrained settings and to calculate the sample size for vasculitis.

Limitations of the study

There are several limitations of this study. As an observational study done in the rheumatology department of a tertiary care hospital, our study may not cover all the cases of vasculitis- and vasculopathy-associated DI in the community. This was a thesis work done during the COVID-19 period, so the total sample could not be enrolled. Duplex study (in four patients) and biopsy could not be done for COVID-19 reasons. All the tests for APS were not possible for patients due to resource constraints. Repeat tests, when required, were not performed because of the study design.

## Conclusions

In a tertiary rheumatology center, vasculitis associated with CTDs is more common in patients with DI. Lupus vasculitis and SSc were the most common causes of DI among the vasculitis and vasculopathy cases, respectively. Patients with PAD can present before the age of 40 years and with ischemia in the upper limb.

## Appendices

Appendix A

Datasheet

Title: Evaluation of patient with digital ischemia for the etiology

Principal Investigator: Dr. Bidhan Neupane

Contact number:

Demographic and clinical characteristics of the patient

1.      Name: …………………………………………………………………………...

2.      Address: ………………………………………………………………………...

3.      Age (years):

4.      Gender: Male=1, Female=2

5.      Occupation: Agriculture=1, Business=2, Service=3, Student=4, Housewife=5, Unemployed=6, Others=7

6.      Education: Illiterate=1, Up to SSC=2, HSC=3, Graduate and above=4

7.      Tobacco use: Yes=1, No=2, Ex-smoker=3

8.      Substance abuse: Yes=1, No=2

9.      Oral contraceptive pills (OCP) intake: Yes=1, No=2, NA=3

10.  Body mass index (BMI) (kg/m2): Underweight=1, Normal=2, Overweight=3, Obese=4

11.  Family history of sudden death: Yes=1, No=2

12.  Autoimmune disease in 1st degree relative: Yes=1, No=2

13.  History of recent trauma: Yes=1, No=2

14.  Disease duration (years):

15.  Fever: Yes=1, No=2

16.  Arthralgia or arthritis: Yes=1, No=2

17.  Myalgia: Yes=1, No=2

18.  Weight loss: Yes=1, No=2

19.  Anemia: Yes=1, No=2

20.  Jaundice: Yes=1, No=2

21.  Cyanosis: Yes=1, No=2

22.  Clubbing: Yes=1, No=2

23.  Edema: Yes=1, No=2

24.  Lymphadenopathy: Yes=1, No=2

25.  Bony tenderness: Yes=1, No=2

26.  Hepatomegaly: Yes=1, No=2

27.  Splenomegaly: Yes=1, No=2

28.  Ascites: Yes=1, No=2

29.  Others:……………………………………………………..……………………………………..……………………………………………………………………………………………………………………………………………………………………………………………………………….

Co-morbidities

30.  Hypertension: Yes=1, No=2

31.  Diabetes or prediabetes: Yes=1, No=2 

32.  Dyslipidemia: Yes=1, No=2

33.  Cardiovascular events (angina or MI): Yes=1, No=2

34.  Stroke: Yes=1, No=2

35.  Thyroid disorder (Hypothyroid or Hyperthyroid): Yes=1, No=2

Investigations

36.  Hemoglobin (g/dL):

37.  Total count (/cumm):

38.  Platelet count (/cumm):

39.  ESR (mm in 1st hour):

40.  CRP (mg/L):

41.  Serum creatinine (mg/dL):

42.  ALT (U/L):

43.  Fasting blood sugar (mmol/L):

44.  2 hours after breakfast blood sugar (mmol/L):

45.  Fasting lipid profile (mg/dl): TC:                HDL:                LDL:                TG:

46.  Urinary albumin: Nil=1, Present=2

47.  Urinary pus cells (/hpf): <5=1, ≥5=2

48.  Urinary RBCs (/hpf): <5=1, ≥5=2

49.  Urinary casts: Present=1, Absent=2

50.  Chest x-ray PA view: Normal=1, Abnormal=2

51.  12 lead ECG: Normal=1, Abnormal=2

52.  Duplex study of limbs: Normal=1, Abnormal=2

………………………………………………………………………………………………

If needed

53.  HbA1c (%):

54.  Anti-centromere Ab: Positive=1, Negative=2

55.  Anti-Scl 70 Ab: Positive=1, Negative=2

56.  Nail-fold capillaroscopy: Normal=1, Abnormal=2

57.  Echocardiography: Normal=1, Abnormal=2

58.  HRCT chest: Normal=1, Abnormal=2

59.  Anti-RNP Ab: Positive=1, Negative=2

60.  ANA: Positive=1, Negative=2

61.  Coombs test: Positive=1, Negative=2

62.  Anti-ds DNA: Positive=1, Negative=2

63.  Anti-Smith Ab: Positive=1, Negative=2

64.  C3: Normal=1, Low=2

65.  C4: Normal=1, Low=2

66.  Lupus anticoagulant: Positive=1, Negative=2

67.  Anti-cardiolipin Ab: Positive=1, Negative=2

68.  Anti-β2GP1 Ab: Positive=1, Negative=2

69.  Anti-Ro/SSA: Positive=1, Negative=2

70.  Anti-La/SSB: Positive=1, Negative=2

71.  Schirmer’s test: Positive=1, Negative=2

72.  Lip biopsy: Normal=1, Abnormal=2

73.  RF: Positive=1, Negative=2

74.  ACPA: Positive=1, Negative=2

75.  CK: Normal=1, High=2

76.  Aldolase: Normal=1, High=2

77.  AST: Normal=1, High=2

78.  LDH: Normal=1, High=2

79.  Anti-Jo-1 Ab: Positive=1, Negative=2

80.  EMG: Normal=1, Abnormal=2

81.  Muscle biopsy: Normal=1, Abnormal=2

82.  pANCA: Positive=1, Negative=2

83.  cANCA: Positive=1, Negative=2

84.  Angiography: Normal=1, Abnormal=2

……………………………………………………………………………………………………………………………………….......………………………………………………………………………………………………………………………………………..

85.  HBsAg: Positive=1, Negative=2

86.     Anti-HCV: Positive=1, Negative=2 

87.     Blood culture and sensitivity: Positive=1, Negative=2

88.     Homocysteine: Normal=1, High=2

89.     Factor V: Normal=1, Low=2

90.     Protein C: Normal=1, Low=2

91.     Protein S: Normal=1, Low=2

92.     Antithrombin III: Normal=1, Low=2

Others: ……………………………………………………………………………………...

………………………………………………………………………………….………...……………………………………………………………………………………………………………….

Classification/Diagnostic Criteria

93.     ACR/EULAR criteria for the classification of systemic sclerosis: Definite=1, Does not fulfil=2 

94.      Diagnostic criteria for mixed connective tissue disease: Fulfil=1, Does not fulfil=2

95.     2019 EULAR/ACR classification criteria for systemic lupus erythematosus: Fulfil=1, Does not fulfil=2

96.     ACR/EULAR classification criteria for primary Sjogren’s syndrome: Fulfil=1, Does not fulfil=2

97.     2010 ACR/EULAR classification criteria for rheumatoid arthritis: Definite=1,          Does not fulfil=2

98.     Revised classification criteria for the antiphospholipid syndrome: Meet=1, Does not meet=2

99.     2017 EULAR/ACR classification criteria for adult and juvenile idiopathic inflammatory myopathies: Definite=1, Probable=2, Does not fulfil=3            

100.    1990 criteria for the classification of giant cell (temporal) arteritis:

Fulfil=1, Does not fulfil=2

101.  1990 criteria for the classification of Takayasu arteritis:

Fulfil=1, Does not fulfil=2

102.  1990 criteria for the classification of polyarteritis nodosa:

Fulfil=1, Does not fulfil=2

103.  Clinical criteria for the diagnosis of Buerger’s disease:

Confident=1, Not confident=2

104.    Diagnostic criteria for infective endocarditis (IE) according to the 2015 ESC guidelines for IE: Fulfil=1, Does not fulfil=2

105.  Features of peripheral arterial disease: Yes=1, No=2

Informed written consent form

Name of participant: …………………………………………………………………….

Study Number: ………………………………………………………………………….

Title: Evaluation of patient with digital ischemia for the etiology

Investigator’s name: Dr. Bidhan Neupane

Institution: BSMMU, Dhaka

Department: Rheumatology

Purpose of the research:

Digital ischemia is an uncommon pathology with incidence of 2 per 100,000 persons per year. It is due to inadequate blood flow to tissues and is one of the clinical manifestations of systemic disease. It has multiple etiologies. Digital ischemia results in disfigurement, functional disability and pain, decreasing quality of life and increasing clinical and financial burden. The patients will be benefited if they are diagnosed and treated early. The results obtained through this study will help in early diagnosis and initiation of treatment plan.

Why the participant is selected for the study:

Patients with digital ischemia are involved in this study. Their participation will help in the early diagnosis and initiation of treatment plan in the sufferers.

What is expected from the participants/respondents:

If you agree to our proposal of enrolling you in this study, we will ask you some questions related to you, your illness and history of taking medication(s), some physical examinations and some relevant laboratory investigations.

Risk and benefits:

There is a minimum physical, psychological, social and legal risk during history taking, physical examinations and investigations.

You will not get any financial support for participating in this study.

Privacy, anonymity and confidentiality:

Data or medical information, like history, examination findings, previous medical records, laboratory reports and description of treatment, identifying you will be maintained strictly confidential. None other than the investigator of this research, possible study guide and any law enforcing agency in the event of necessity will have an access to the information. Data related to the study may be sent outside the country for analysis where applicable. However, any personal identifiable information will be held and processed under secured conditions, with access to limited appropriate staff of that organization. We would be obliged to provide you the information related to medical conditions of your treatment or results of any or all tests performed on you. You would be able to communicate freely with the investigator of this study.

Future use of information:

In case of future use of the information collected from this study, anonymous or abstracted information and data may be supplied to other researcher(s). This will not conflict with or violate maintenance of privacy, anonymity and confidentiality of information identifying the participants in any way.

Right not to participate or withdraw:

Your participation in this study is voluntary. You are the sole authority to decide for and against your participation. Your medical care will not be hampered even if you do not enroll in this study.

Principle of compensation:

Your participation in this study will be considered as voluntary and no compensation will be provided. For any query or information about the rights or benefits of your involvement in this study, you are free to contact with us.

Dr. Bidhan Neupane

Department of Rheumatology

Bangabandhu Sheikh Mujib Medical University, Dhaka.

Phone number- 01768279952

If you agree to our proposal of enrolling you in the study, please indicate that by putting your signature or your left thumb impression.

Thank you for your cooperation.

Signature or left thumb impression of the participant                           Date                           

Signature or left thumb impression of the witness                               Date               
